# A machine learning approach for type 2 diabetes diagnosis and prognosis using tailored heterogeneous feature subsets

**DOI:** 10.1007/s11517-025-03355-5

**Published:** 2025-04-08

**Authors:** J. Ramón Navarro-Cerdán, Pedro Pons-Suñer, Laura Arnal, Joaquim Arlandis, Rafael Llobet, Juan-Carlos Perez-Cortes, Francisco Lara-Hernández, Celeste Moya-Valera, Maria Elena Quiroz-Rodriguez, Gemma Rojo-Martinez, Sergio Valdés, Eduard Montanya, Alfonso L. Calle-Pascual, Josep Franch-Nadal, Elias Delgado, Luis Castaño, Ana-Bárbara García-García, Felipe Javier Chaves

**Affiliations:** 1 Universitat Politècnica de València, Camí de Vera, s/n, 46022 València, Spain; 2 ITI, Universitat Politècnica de València, Camino de Vera s/n, 46022 València, Spain; 3 Genomic and Diabetes Unit, INCLIVA Biomedical Research Institute, 46010 València, Spain; 4 CIBERDEM, ISCIII, Madrid, Spain; 5 UGC Endocrinología y Nutrición, Hospital regional Universitario de Málaga, Instituto de Investigación Biomédica de Málaga y Plataforma en Nanomedicina-IBIMA Plataforma BIONAND, Málaga, Spain; 6 Bellvitge Hospital-IDIBELL, Barcelona, Spain; 7 Department of Clinical Sciences, Barcelona, Spain; 8 Medical School, University Complutense, Madrid, Spain; 9 Endocrinology and Nutrition Department, Hospital Clínico Universitario San Carlos, Madrid, Spain; 10 EAP Raval Sud, Catalan Institute of Health, GEDAPS Network, Primary Care, Research Support Unit (IDIAP-Jordi Gol Foundation), Barcelona, Spain; 11 Department of Endocrinology and Nutrition, Central University Hospital of Asturias, Health Research Institute of the Principality of Asturias, Oviedo, Spain; 12 CIBERER, Madrid, Spain; 13 Cruces University Hospital, Biocruces Bizkaia Health Research Institute, Endo-ERN, UPV/EHU, Barakaldo, Spain; 14 ITI, Instituto Tecnológico de Informática, Camino de Vera s/n, 46022 València, Spain

**Keywords:** Type 2 diabetes mellitus, Geospatial data augmentation, Quasi-constancy heuristic, Heterogeneous missing data imputation, Feature selection, Diagnosis and prognosis risk estimation

## Abstract

**Abstract:**

Type 2 diabetes (T2D) is becoming one of the leading health problems in Western societies, diminishing quality of life and consuming a significant share of healthcare resources. This study presents machine learning models for T2D diagnosis and prognosis, developed using heterogeneous data from a Spanish population dataset (Di@bet.es study). The models were trained exclusively on individuals classified as controls and undiagnosed diabetics, ensuring that the results are not influenced by treatment effects or behavioral changes due to disease awareness. Two data domains are considered: environmental (patient lifestyle questionnaires and measurements) and clinical (biochemical and anthropometric measurements). The preprocessing pipeline consists of four key steps: geospatial data extraction, feature engineering, missing data imputation, and quasi-constancy filtering. Two working scenarios (Environmental and Healthcare) are defined based on the features used, and applied to two targets (diagnosis and prognosis), resulting in four distinct models. The feature subsets that best predict the target have been identified based on permutation importance and sequential backward selection, reducing the number of features and, consequently, the cost of predictions. In the Environmental scenario, models achieved an AUROC of 0.86 for diagnosis and 0.82 for prognosis. The Healthcare scenario performed better, with an AUROC of 0.96 for diagnosis and 0.88 for prognosis. A partial dependence analysis of the most relevant features is also presented. An online demo page showcasing the Environmental and Healthcare T2D prognosis models is available upon request.

**Graphic abstract:**

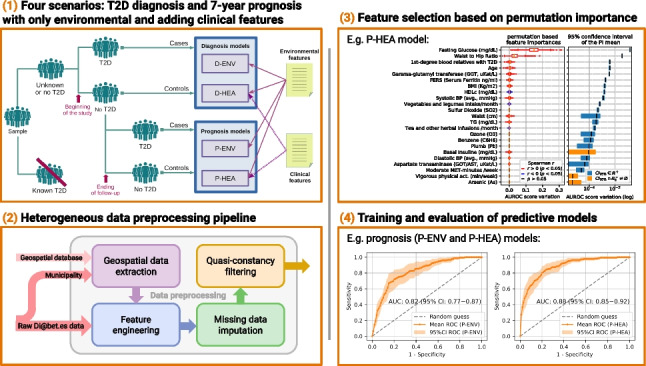

**Supplementary Information:**

The online version contains supplementary material available at 10.1007/s11517-025-03355-5.

## Introduction

Diabetes is a metabolic disease that affects how the body uses or produces insulin, thereby affecting blood glucose levels. It leads to a range of health problems, such as vision loss, stroke, renal failure, heart attacks, or lower limb amputation. In 1990, about 300 million people suffered from diabetes worldwide; by 2021, this number had risen to about 529 million people; and by 2050, it is expected to exceed 1.3 billion [1, 2]. Type 2 diabetes (T2D) cases account for 96.0% (95% confidence interval (CI) 95.1–96.8) of all diabetes cases [2]. Additionally, over the last two decades, there has been a 3% increase in age-standardized mortality rates from diabetes, while mortality rates are decreasing in the main non-communicable diseases [1]. In the Spanish population, up to 14% of people suffer from T2D, and this proportion is increasing. Moreover, nearly half of these individuals are unaware that they have the disease (6.0% [5.4, 6.7%]) [3, 4].

T2D can be diagnosed using various biochemical parameters such as fasting plasma glucose, haemoglobin A1C or 2-hour plasma glucose during an oral glucose tolerance test (OGTT) [5]. However, many individuals with T2D remain undiagnosed: its symptoms could be mild, causing the delay of the diagnosis after complications have arisen [1]. Determining risk factors that are easy to obtain for risk estimation, with or without biochemical analysis, can help to reduce the number of undiagnosed T2D patients by identifying those at higher risk. Prevention and early diagnosis of T2D leads to significant reductions in mortality, improvements in quality of life, and decreased healthcare costs worldwide [6]. Therefore, improving customized risk calculation would enhance prevention programs by targeting individuals with higher risk. At the same time, it would also provide insights into the disease by identifying new risk factors.

T2D is a complex disease with many factors involved in its development. High Body Mass Index (BMI) was the primary risk factor for T2D worldwide, accounting for over 50% of global T2D [1, 2]. Other risk factors have been identified and can be related to this primary risk factor (dietary, occupational, environmental, physical activity, smoking, and alcohol consumption) [1, 2], although many other factors can also influence T2D development. Thus, T2D requires complex models to achieve the best performances when attempting to predict the risk of its appearance. AI has significant potential to improve T2D diagnostic and prognosis, as evidenced by recent surveys on this topic [7, 8].

Potential uses of T2D diagnosis and prognosis models include risk stratification and prevention plans, although more integration needs to be reported in the literature. The survey presented in [7] points out that only 12 out of the 23 analyzed studies developed any form of prediction tool (nomogram, risk score, decision rules, or screening tools). A notable study is presented in [9], where models have been integrated into a publicly available online tool and used within the UK National Health System.

In this work, data from individuals across Spain with baseline information (gathered at the start of the study) related to T2D and a 7-year follow-up have been used. This includes heterogeneous clinical and environmental features. The study covers several stages: initially, extensive work is done on various techniques and heuristics to enhance data quality; subsequently, the enhanced data is used to identify relevant factors related to T2D; and finally, T2D diagnosis and prognosis models are built and tested, combining diverse groups of factors.

The main contributions of this study are machine learning-based predictive models for T2D diagnosis and prognosis that could be used as part of a decision support tool. Furthermore, the results obtained in this work encompass the identification of relevant factors and the satisfactory prediction performances achieved by incorporating environmental and clinical features together. Prognosis models can be tested via an online demo.

The remainder of this paper is structured as follows: a brief literature review is presented next. Following this, the Materials and methods section spans several subsections: description of the data used in the study (Dataset and Dataset extension with geospatial data sections), the data quality improvement pipeline (Feature engineering, Missing data imputation, and Quasi-constancy filtering sections), the experimental design, feature selection pipeline (Feature importance and Feature selection sections), model training and evaluation and explainability via partial dependency analysis. The results of the aforementioned steps are described and discussed in the Results and Discussion sections. Finally, the Conclusion section outlines the main conclusions of this work and future work.

### Literature review

Previous studies have shown performances greater than 0.9 Area Under the Receiver Operating Characteristic Curve (AUROC) in diagnosis [10] by combining random forest and logistic regression classifiers. Some works have achieved a score close to 1 AUROC, although with small sample sizes [11] or including blood glucose (a direct diagnostic measure for diabetes) as a predictor [12]. Even though prognosis models have reported lower performance, they are still high nonetheless. For instance, authors in [13] trained a multiple instance boosting algorithm to predict the worsening of insulin resistance over nine years, achieving an AUROC of 0.89. Another approach is to have multiple models, depending on the difficulty level of obtaining the answers or test results required for its use. Nanri et al. [14] followed this approach to predict the 3-year incidence of T2D, reporting 0.734 AUROC for the non-invasive model and 0.882 AUROC for the invasive model, both in an independent validation cohort.

**Table 1:** Summary of related work from various studies on T2D prediction, following the columns proposed in [10]

Ref	Year	Dataset	Data size	Feat. selection	Classifiers	Accuracy	AUROC	Task	Protocol
[10]	2020	NHANES study	6561	LR	RF	0.94	0.95	Diagnosis	10-Fold
[11]	2020	EMRP-107-048 Study	149	IG	XGB,RF,SVM, C5.0,DNN	1.0	1.0	Diagnosis	10-Fold
[12]	2020	HOSP FRANKFURT (Kaggle)	2000	ADABOOST, RF,DT	DT	0.99	0.998	Diagnosis	10-Fold
[13]	2020	FIMMG	256	–	GBT,DT, RF,SVM	0.87	0.94	9-year incidence	10-Fold
[14]	2015	J-ECOH study	37416	BACKWARD	LR	–	0.88	3-year incidence	Train (24950) Test (12466)
[17]	2022	Di@bet.es study	2570	CHAID	DT	0.935	–	7.5-year incidence	K-Fold
[18]	2003	FINRISK	4595	LR	FINDRISC	–	0.857	Prognosis	–
[18]	2003	FINRISK	4435	LR	FINDRISC	–	0.860	Prognosis	–
[19]	2011	Pizarra Study	1051	–	FINDRISC	–	0.74	Diagnosis	–
[19]	2011	Pizarra Study	824	–	FINDRISC	–	0.75	6-year incidence	
[20]	2016	Northern Colombia	2060	–	FINDRISC	.	0.74	Diagnosis	–
[20]	2016	Northern Colombia	2060	–	FINDRISC	.	0.70	IGR prognosis	–
[21]	2018	North Peru	1609	–	FINDRISC	.	0.69	Diagnosis	–

(*LR* logistic regression, *GBT* gradient boosting trees, *RF* random forest, *XGB* extreme gradient boosting, *SVM* support vector machine, *IGR* impaired glucose regulation, *IG* information gain, *CHAID* Chi-squared automatic interaction detection)

It is worth highlighting the variability between results from different studies, likely due to the heterogeneity of the datasets (for instance, in terms of variables, population, or sample sizes). Moreover, most datasets are private, further hindering the comparison between models. Some well-known public datasets for diabetes diagnosis include the Pima Indians Diabetes Dataset, containing information of 768 women and nine features, and the Mendeley Diabetes Dataset, containing 1000 patients and 11 features [15, 16]. There are different studies using machine learning algorithms, and most of them show differences in sample size, classifiers, protocol types, and accuracy. Table 1 presents a summary of the methodologies and performance results from various studies on T2D prediction, following the columns proposed in [10]. Sample sizes vary from about 750 to 37.000 samples, including both individuals with and without T2D, but typically considering only diagnosed patients. In addition, most studies analyze all available variables together without accounting for their source or specific utility [10–14]. In this context, using parameters that can be self-assessed by individuals may be beneficial for detecting at-risk populations, both present and future. Meanwhile, biochemical parameters directly involved in T2D diagnosis (such as glucose levels or glycated hemoglobin), as well as other parameters closely related to T2D like triglycerides (TG) and total cholesterol, can be useful in the direct diagnosis of the disease and for predicting its future development [5, 17].

Several works in the past focused on selecting the most informative features related to T2D development, aiming to demonstrate that responses from easy-to-fill forms can provide a basic assessment of the risk of developing T2D. These basic models can be made available to a broad population at minimal costs. FINDRISC (Finnish Diabetes Risk Score) [18] is one of these popular tools worldwide. It includes only eight features encompassing anthropometric and lifestyle factors that most people can readily answer to get an instant evaluation. It does not involve blood tests or any other specific medical test so that it can be used as a low-cost screening tool. This simple questionnaire can identify individuals at high risk of developing T2D within the next ten years with, generally, high accuracy. In [19], the authors used FINDRISC on a local population in Spain and predicted undiagnosed T2D with 0.74 AUROC and incident T2D with 0.75 AUROC. FINDRISC assessments in other countries include Colombia (AUROC: 0.73) [20], Peru (AUROC: 0.69) [21], and other systematic studies carried out in Latin America (AUROC consistently above 0.65) [22]. These and many other studies have used and compared questionnaires with similar approaches across different populations, usually achieving good performance, especially considering the small number of low-cost features required.

## Materials and methods

This section explains the experimental design. First, we describe the dataset used and the preprocessing performed on it. Then, feature selection, model training, and evaluation are detailed. Lastly, partial dependence analysis is described.

### Dataset

The dataset used consists of 242 environmental features extracted from 4617 anonymized individuals from Di@bet.es, a study that examines the prevalence of diabetes and impaired glucose regulation among the Spanish population [3]. This dataset gathers baseline information from all the individuals at the beginning of the study, as well as follow-up information throughout $7.48\pm 0.55$ years from 1850 of the participants. Regarding the target task, the dataset contains information of each individual on: 1) whether the individual had been previously diagnosed with T2D, 2) whether the individual was diagnosed at the beginning of the Di@bet.es study (based on the clinical information gathered), and 3) if included in the follow-up, whether the individual developed T2D during the follow-up period.

The features available in the Di@bet.es dataset were classified and grouped into two domains, following the taxonomy shown in Table 2. These two domains, Environmental (ENV) and Clinical (CLI), can be associated to different costs of acquisition, for instance, it is assumed that having a patient fill out a questionnaire is easier and cheaper than performing a blood test.

**Table 2:** Taxonomy of the features available in the Di@bet.es dataset

Domain	Feature type	Source
ENV	Lifestyle, family historyAnthropometry, age, sexDiseases and treatmentsGeospatial	Patient questioningPatient measuringMedical historyOfficial databases
CLI	Clinical-biochemistry	Biological analysis

Five types of features have been considered depending on the source they came from. Heterogeneity is managed by grouping all of them in two domains

**Fig. 1:**
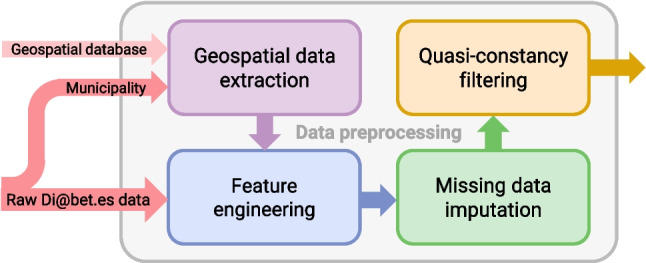
Main sequence of processing steps applied to all data. Feature-dependent processes were applied, such as removing redundancy among features, data imputation using univariate or multivariate methods, and quasi-constancy filtering

The raw data exhibited several issues affecting the quality of the features, such as missing values, features with very low variability (quasi-constancy), string errors, and redundancy. Three processes for data quality improvement and feature extraction were applied to all the data: feature engineering (string correction and feature grouping), missing data imputation, and quasi-constancy filtering. Additionally, a geospatial data extraction process (generating features derived from the municipality, in this case, related to pollution and income) complemented the dataset with new features. Figure 1 represents the flow of these processes,^1^ which are described in the following subsections.

### Dataset extension with geospatial data

We hypothesized that certain features related to a patient’s living area could influence the development of T2D. Therefore, we leveraged municipality records to augment our dataset with additional information derived from ancillary databases. Specifically, we extended our dataset with two demographic features, *Population* and *Income per consumption unit*,^2^ and with twelve pollution features (previously used in [23]). We used *Municipality* field from Di@bet.es dataset to link each patient to its corresponding geospatial data. Since *Municipality* strings were manually collected, we applied automatic error correction and standardization using a weighted finite state transducer composition technique [24]. The list of all geospatial variables along with associated short descriptions, can be found in Supplementary Material 1, Section 1, Table S2.

The consulted ancillary database provided pollution information from 381 municipalities gathered through geographical sensors [25]. For each patient, we retrieved the pollution data from the preceding 10 years in their area, based on the date of each observation acquisition. To aggregate this data, we computed the weighted moving average ($\bar{x}_{w}$) as shown in Expression (1), where the last years are assigned higher weight, which also worked to smooth single-year peaks and casual inaccurate measurements.

1 $$\begin{aligned} \bar{x}_w = \sum ^{n=10}_{i=1}\frac{i \cdot x_{i}}{w} \text{, } \text{ where } w = \frac{n(n+1)}{2} \end{aligned}$$

**Fig. 2:**
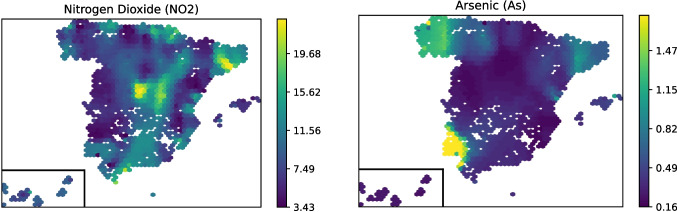
Example of geospatial features: $NO_2$ (left) and *As* (right) pollution in the Spain territory. Geospatial features are fully imputed through an interpolation of the available external geospatial data [25] using Expression (2)

A substantial fraction of the dataset (50.4%) lacked pollution values, given that only 56 of 319 non-duplicate municipalities found in Di@bet.es had a registry in the geospatial database. To address this issue, we planned to impute those missing values based on an elasticity model, as defined in Expression (2). Let $f_k$ be a feature associated with the municipality *k*, let *d*(*m*, *k*) denote the Euclidean distance between municipalities *m* and *k*, and let $\beta$ be the scaling parameter that accounts for elasticity between municipalities. The value $\hat{f}_m$ imputed to a municipality *m* as a function of its *k*-nearest municipalities is computed as follows:

2 $$\begin{aligned} \hat{f}_{m} = \sum \limits _{i=1}^k\dfrac{w^m_{i} f_{i}}{\sum \limits _{j=1}^k w^m_{j}}~, ~~w^m_{l} = \dfrac{1}{d(m,l)^\beta } \end{aligned}$$

where $\hat{f}_{m}$ represents the inverse-distance-weighted average of the $k-$nearest measurements [26], which ensures that closer points contribute more significantly. In this work, to estimate pollution values, we set $\beta =2.0$, which quadratically penalizes the distances. Since an accurate estimation of this parameter could require very complex models, we based our decision on commonly used estimations for this kind of models and visual checking of the maps instead. We also set $k=10$, meaning that each imputation considers the 10-nearest measurements. Figure 2 shows an example of two pollution measurements (*Nitrogen dioxide* (${NO_{2}}$) and *arsenic* (*As*)) after imputation across Spain. All 14 pollution metrics, as well as average income and population, are listed in Supplementary Material 1, Section 1, Table S2.

The mean income per consumption unit for each location was extracted from the INE database [27]. Approximately 20% of these values were missing, and they were also imputed using Expression (2). *Population* values were available for all municipalities and could be directly assigned to all observations in the study.

In order to mitigate the risk of introducing biases from geospatial data, individuals residing in the same region^3^ were not distributed across both training and test sets within the same data splits in subsequent experiments. An example of the sort of bias we aim to prevent occurs when a pollution value is particularly low or high in a region with an illness incidence different from the baseline, although the cause for this is not said to be pollution but another unknown factor. Even though we removed the municipality feature from the dataset, the model could still learn to identify individuals living in this region based on the localized pollution data anomalies, potentially leading to false attributions of causal relations with T2D development. Using the municipality attribute that was extracted from the dataset, we assigned a region label to each individual, creating groups that are kept together during dataset splits. This approach ensures that any bias learned during training would not manifest as apparent improvements in test metrics, thereby preventing misidentification of features as relevant due to unknown regional effects. Bias limitations of these techniques could arise for individuals from neighboring regions that are actually closer.

Our models did not include the *Municipality* (town or city of residence) feature, but the geospatial features associated. This was intended to generalize the use and conclusions of this work, not restricted them to Spain.

### Feature engineering

Redundancy refers to features sharing partial or complete meaning or effect on the target. Redundancy can distort the results of a study at different levels. For example, the importance of redundant informative features could be distributed among them during a feature selection process, possibly making them harder to identify. Additionally, they can add difficulty or distort the interpretability of explainability methods.

Redundancy among features is frequent in medical datasets, particularly those derived from patient questionnaires, measurements, or medical history. An example of redundancy affecting the Di@bet.es dataset is the presence of the features *bread ingestion*, *rice ingestion*, and *pasta ingestion*, which could be combined into a single representative feature: *carbohydrate-rich food ingestion*. Other examples include derived features, such as *obesity* or ${BMI>=30}$, which is derived from *BMI*.

All derived features resulting from thresholding another feature were removed. Features derived from the body mass index (*BMI*), such as ${BMI>=30}$ and ${BMI<=19}$, were discarded, retaining only the raw *BMI* since it contains more detailed information. Similarly, features based on thresholding *waist circumference* were also removed, keeping only the original measurement in centimeters. While *waist-to-hip* ratio is a handcrafted feature calculated by dividing *waist circumference* by *hip circumference*, the two latter variables cannot be deduced only from the resulting division. The combination of both is not linear nor based on a simple threshold and could contribute additional information, while the individual variables may remain relevant on their own.

According to physicians and biologists’ expertise, many features included in the Di@bet.es study have very similar semantics and could have very similar effects on T2D development. While these sets of similar features are not necessarily correlated, they could benefit from being combined into more representative features or undergoing different feature extraction transformations. Based on this knowledge, particular sets of variables were grouped by semantics and used to construct new variables. Following this, the original features were removed from the study. This helped to reduce the sparsity of variables in the dataset. Furthermore, the link between the new, more representative features and T2D development could be more easily recognized than with their former, more sparse components.

The following list outlines additional feature extraction processes performed on the Di@bet.es database, leveraging domain expertise. Many of the affected features are related to eating habits, obtained through a food frequency questionnaire. For each item, patients reported their intake as a daily, weekly, or monthly count. For convenience and to ensure consistency, all responses were standardized to weekly counts.

- We found that many features were related to smoking habits. The questions addressing this topic (*Since when have you smoked*, *Number of cigarettes per day*, *Did you quit*, and *When did you quit*) were used to derive the new feature *Smoking pack-years*. This feature was calculated by multiplying the number of years the patient smoked by the estimated number of cigarette packs consumed per day. Although some patients quit smoking before the study, the *Smoking pack-years* variable could provide insights into the potential long-lasting effects of smoking. Additionally, the response to the question *Do you currently smoke* was retained as an individual feature, as whether or not the patient currently smokes could hold distinct relevance.
- Intake of tea and other different kinds of herbal infusions per month were combined into a single feature.
- Features measuring the intake of different kinds of coffee were merged into a single *coffee consumption* feature. Furthermore, caffeinated beverages, including applicable types of coffee, were combined into a single *caffeinated beverages per month* feature. While these two features are correlated, their unique information justifies the inclusion of both.
- Features related to the consumption of different wines were combined into a single *wine consumption* feature. Similarly, features related to both wine and other alcoholic beverages were aggregated into a single *alcohol consumption* feature. Note that, although wine consumption is expected to be correlated with alcohol consumption, we considered that wine may have a specific effect on T2D, as it is distinct from other alcoholic beverages, while alcohol consumption itself could influence T2D, independently of the type of drink.
- The intake of different vegetable-based sides, salads, and legumes was summed into a new *vegetables and legumes intake* feature.
- Consumption of fried, pre-cooked meals, and bag snacks were combined into *fried and precooked food intake*.
- Intake counts of dairy products, eggs, and other animal-based non-meat protein sources were combined into *eggs and dairy intake*.
- Combining the intake of products rich in carbohydrates, such as bread, rice, pasta, and potatoes, we created a single *carbohydrate-rich food ingestion* feature. However, information regarding the kind of grain (white or whole-grain) of some of these products was preserved in the new *% whole-grain products* feature, ranging from 0 to 1, where the maximum indicates whole-grain product intake only.
- Foods high in simple sugar content, such as chocolate, jam, honey, candies, and some desserts, were combined to create the *sugar snacks and desserts intake* feature.
- Consumption of sides and sauces rich in fats, such as margarine, mayo, and butter, was merged into the *fatty sides intake* feature.

### Missing data imputation

**Fig. 3:**
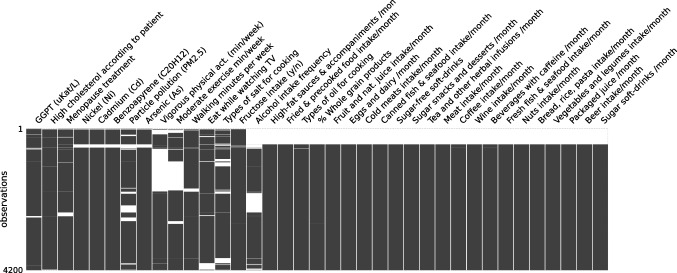
Missingness matrix of the Di@bet.es dataset after performing feature engineering. Each column represents a feature, and each row represents an observation. White spaces indicate missing values, while gray color represents observed values. Only features with a missingness fraction equal or greater than 1% are displayed. Rows were reordered using hierarchical clustering based on the presence of missing values to enhance the visualization of potential missingness patterns or correlations among features

The amount and patterns of missing values in the dataset vary significantly across variables. Figure 3 shows the distribution of missing data for all features with at least 1% missing values. Several multivariate missingness patterns are evident. The missingness of geospatial variables in the dataset is consistent across individuals, as these variables are solely linked to the municipality, i.e., if this link is missing, values for the geospatial features cannot be directly assigned. Similarly, features related to vigorous and moderate exercise exhibit monotone missingness. Also, missingness is consistent across many features related to eating habits, likely reflecting whether patients responded to the food frequency questionnaire.

Different types of imputation were applied to address this issue in different variables. Univariate methods fill in missing values within a specific variable based on information from other observations within that same variable. Alternatively, multivariate methods leverage the information from other variables of the same individual, learning from the relationships observed in other individuals with complete entries.

**Table 3:** Imputers used and their type

Imputer	Univariate	Multivariate
Median [29]	$\checkmark$	
Most_frequent [29]	$\checkmark$	
Mean [29]	$\checkmark$	
APP random$^1$[28]	$\checkmark$	
k-NN (k=5) [30]		$\checkmark$
RF Iterative [31, 32]		$\checkmark$

($^1$) It preserves the original distribution

In this work, we used the IQA algorithm [28] to select the best imputer for each variable among the set of well-known imputation algorithms shown in Table 3. The IQA algorithm evaluates a range of imputers and selects the most appropriate one for each feature, taking into consideration the potential biases that can be introduced during the imputation process. It employs statistical tests to reject any imputation that would make imputed values easily discernible. The optimal imputer for each variable is determined based on a Quality Score (QS) defined in Eq. 3. This score is calculated as a combination of the initial completeness of the variable and the performance of an imputer that fills its missing values based on available complete data. This way, a variable with no missing values achieves the maximum score (100%), as does any variable with some missing values that can be accurately imputed without error. Variables that cannot reach a minimum quality score of 0.7 due to high incompleteness or unreliable imputation were excluded from further analysis.

3 $$\begin{aligned} \text {QS} = \gamma +(1-\gamma )*\mu \end{aligned}$$

where:

$$\begin{aligned} \gamma&= \text {\% of complete entries in the column}, [0-1]\\ \mu&= \text {imputation score, e.g.}~R^2~\text {score}, [0-1] \end{aligned}$$

Preparing data for imputation, as well as deciding between a classifier or a regressor for the corresponding imputer, makes it necessary to distinguish *a priori* between nominal and quantitative variables. To consider a variable as nominal, a minimum appearance frequency of 5 of every unique value was required, as recommended by [33] for a $\chi ^2$ test. Once the nature of the variables was established, the nominal explanatory variables were hot-encoded.

In the case of multivariate imputation techniques, the imputers were always trained using observations from the training sets and then applied to both train and test sets for filling missing values. This ensures consistency and prevents over-optimistic model performances in subsequent stages. A comprehensive list of variables and the imputers applied to each one is given in Supplementary Material 1, Section 2.

### Quasi-constancy filtering

Features with very low variability are commonly referred to as *quasi-constants*. Quasi-constancy can adversely affect classifiers, particularly in the presence of imbalanced data, and may mislead feature selection algorithms into selecting these as informative. Therefore, filtering out quasi-constant features before classification can be a crucial quality-driven step. In this work, we used a quasi-constancy filter based on two metrics: the coefficient of variation [34] and the Gini coefficient [35]. Both indexes range from 0 (indicating constant features) to 1 (indicating the highest variability).

The coefficient of variation is traditionally defined as the ratio of the standard deviation to the mean. Here, we introduce a transformation $\mathcal {L}$ that relates the standard deviation to each observation value instead. Let $x=(x_1,x_2,\dots ,x_n) \in \mathbb {R}^+$ be a n-dimensional predictive variable that has been standardized and shifted to the real positive domain, where the corresponding standard deviation is denoted by $\sigma _{x}$, the transformation $\mathcal {L}$ is defined as follows:

4 $$\begin{aligned} \mathcal {L}(x)= \left( s\frac{\sigma _{x}}{x_{1}}, s\frac{\sigma _{x}}{x_{2}}, \cdots , s\frac{\sigma _{x}}{x_{n}}\right) \text {, where}\hspace{5pt} s=\frac{\overline{x}-\widetilde{x}}{|\overline{x}-\widetilde{x}|} \end{aligned}$$

Here, *s* is used to transform negative skewed distributions into positive ones. The $\mathcal {L}$ transformation yields a series of dimensionless values. Subsequently, the Gini coefficient $\mathcal {G} \in \left[ 0,1\right]$ is applied to the values of $\mathcal {L}(x)$ to measure their inequality. $\mathcal {G}(\mathcal {L}(x))$ serves as a quasi-constancy index, which tends to increase with higher variability and entropy of *x*, and tends towards 0 when *x* is nearly constant (low entropy).

In our experiments, we set a threshold at $\mathcal {G}=0.05$, discarding variables with indices below this value. This threshold was empirically determined through general simulations, where the quasi-constancy value of the least relevant feature in regression problems was measured across various scenarios using synthetic datasets of different sizes. A more detailed summary of this process is provided in Supplementary Material 1, Section 3: “Quasi-constancy index of dataset features.”

### Experimental design

Two scenarios are considered assuming that different types of features can be available in practice. These scenarios are defined by distinct feature domains, each one encompassing specific sets of features:

- *Environmental* (ENV). This includes features of the Environmental domain. Prediction models that only use these kinds of features should be considered low-cost in terms of the effort required to gather the features, their ready availability, and economic factors.
- *Healthcare* (HEA). This includes features from both Environmental and Clinical domains. It may be considered as having medium cost as it needs common clinical procedures such as blood tests in addition to features of the Environmental domain.

After performing the data preprocessing steps, the number of features available for experimentation is shown in Table 4. The ENV scenario comprises 84 features, while the HEA scenario includes 100 features. Notably, the Environmental domain features are included in both scenarios, meaning that the features in the ENV scenario are a subset of those in the HEA scenario.

**Table 4:** Number of features available for experimentation

Domain	Feature type	#Features	
Environmental	Lifestyle, family historyAnthropometric, age, sex Diseases and treatmentsGeospatial	5181114	84
Clinical	Clinical-biochemistry	16	16

**Fig. 4:**
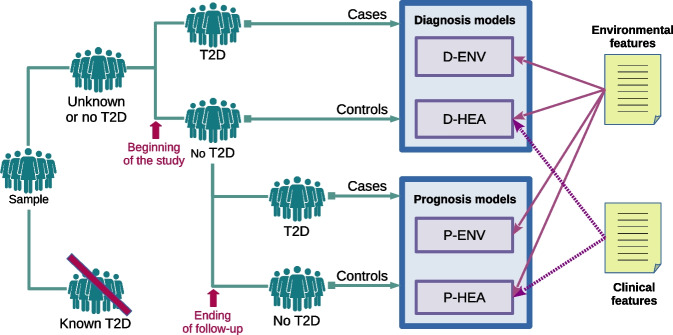
Models for diagnosis (D) and prognosis (P) are built for each scenario (ENV, HEA). The figure outlines the individuals and features that take part in each model

In this work, models for diagnosis (D) and prognosis (P) were trained and assessed for each scenario (ENV, HEA), resulting in four models: D-ENV, D-HEA, P-ENV, and P-HEA. Figure 4 outlines the individuals and features that take part in each model. Individuals diagnosed with T2D before the study (“known T2D”) were excluded from all scenarios to prevent any bias from lifestyle and treatment adjustments typically made after a diagnosis. As a result, the dataset for diagnosis models’ training and evaluation includes 4200 individuals (“unknown or no T2D”).

For the diagnosis models, individuals are assigned to a class (case or control) whether they are diagnosed with T2D for the first time at the beginning of the study (5.4%), or not. The explanatory variables used are the baseline values (measurements at the beginning of the study).

For the prognosis model, individuals diagnosed with T2D at the start of the study and those lacking follow-up variables were excluded. The remaining 1608 individuals were assigned to a class at the end of the follow-up depending on whether they had developed T2D at any moment during the 7-year period (7.8%), or not. As with the diagnosis models, the explanatory variables are the baseline values. Thus, follow-up data were not included, as the intended use is to predict if an individual will develop the disease in the future given their current status.

To obtain the best subsets of predictors and construct the prediction models, a suitable classification algorithm must be chosen. In [36], the authors carried out a comparative empirical study on 11 well-established classifiers across 71 datasets sourcing from various domains. In this study, the Stochastic Gradient Boosting Trees (GBDT) algorithm showed the best performance in terms of mean AUROC. Meanwhile, in [37], the authors compared state-of-the-art gradient boosting frameworks (i.e., XGBoost, LightGBM, and CatBoost) and the classic GBDT algorithm on several diverse, publicly available real-world datasets. They concluded that all considered variants of gradient boosting perform exceptionally well, with each being the best choice in different scenarios. In light of these studies, and considering the widespread adoption, popularity, and extensive documentation of XGBoost, we decided to use the XGBoost algorithm in this study. XGBoost offers a highly scalable end-to-end tree boosting system that is faster and has improved regularization compared to other popular implementations of the original GBDT algorithm [38]. Furthermore, XGBoost is easier to optimize on tabular data than deep neural networks, another state-of-the-art approach that is gaining popularity and may surpass GBDT in some scenarios [39].

### Feature importance

The feature selection process carried out consists of two consecutive phases. In the first phase, we compute the feature importance and provide a ranking of the most important features for each scenario and task. The second phase selects the most appropriate subset for each predictor. This section describes the method used in this first phase, while the next section tackles the second phase. In both phases, we obtained results for each of the four model settings described in Sect. 2.6 using an XGBoost classifier as an estimator, with the same hyperparameters used in model training.

To compute the feature importance, we used the widely known variable permutation technique Permutation Importance (PI) [40]. This method involves several stages. In the first, a model is trained and evaluated with the original data, and its AUROC is computed. Following this, the values of each feature are shuffled one at a time, and the AUROC is computed again for each permutation. The “AUROC score variation” is calculated as the drop in the score after permuting each feature. Based on this variation, the features are then ranked in order of importance. Following these steps, we obtained four different lists (one for each model: D-ENV, D-HEA, P-ENV, P-HEA). The hypothesis is that features having the highest AUROC score variation (greater performance loss) are the most important. It is worth mentioning that PI not only considers single variable effects but also automatically takes into account possible interactions between variables, provided that the model used has found them during its training. This is due to the fact that permuting the values within a single variable destroys not only its relationship with the target but also the interaction effects with all other variables [41]. Although the interaction effect between a pair of variables will be redundantly accounted for in both, without showing how much the interaction contributes to each, this ensures that no variables are discarded in later stages due to them not having a direct effect on the target on their own, thus helping to preserve valuable interactions.

We ran 100 repetitions, each of them randomly splitting the sample into training (70%) and test (30%) sets. Using the results from these multiple repetitions, we calculated the 95% confidence interval of the mean permutation importance for each feature. If a feature’s 95% CI does not overlap with zero or negative numbers (in other words, it pertains only to $\mathbb {R}^+$), we consider that it has a significant and positive contribution to the model’s performance. The features that meet this condition are the starting point for the subsequent feature selection process, which is explained in the next section.

### Feature selection

Once we identified the features that contribute significantly to each model’s performance using the methodology described in Sect. 2.7, we applied a feature selection algorithm to remove the features that did not contribute meaningfully to predictions. For feature selection, we ran a sequential backward selection [42], i.e., a sequential feature search (SFS) that only involves backward steps. The process begins with the set of important features identified in the feature importance phase. At each step, one feature is removed, resulting in a new subset with one less feature. The same splitting strategy used in the feature importance phase (training and validating 100 models) was also used here. At each given step, the feature that, when removed, yields the best average score is the one chosen for elimination. This process continues until only one feature remains. Finally, the scores obtained at each step are ranked, and the subset of features that received the highest score is selected to train the prediction model.

### Model training and evaluation

Consistent with previous experiments, the four proposed models, Diagnosis - Environmental (D-ENV), Diagnosis - Healthcare (D-HEA), Prognosis - Environmental (P-ENV), and Prognosis - Healthcare (P-HEA), were trained using an XGBoost classifier on their respective samples and selected features. A hyperparameter optimization was performed for each model through a 20-fold cross-validation-based search, using Tree Parzen Estimator (TPE) [43], a widely-used versatile Bayesian optimization method. Additionally, to address the class imbalance, the XGBoost algorithm’s parameter *scale pos weight*, which allows for increasing the weighting of observations of the positive class, was also empirically adjusted during hyperparameter tuning to maximize AUROC. Readers can find a summary of the explored hyperparameter search spaces in Supplementary Material 1, Section 5: “Model hyperparameters.”

After fitting the models, their performance score (mean AUROC and 95% confidence interval of the AUROC mean) is calculated through 10-fold cross-validation.

### Feature partial dependence analysis

Model explainability can be addressed by means of partial dependence estimations [44]. A partial dependence plot (PDP) illustrates the marginal effect one feature has on the predicted outcome [45], i.e., the amount a feature contributes to the overall prediction by itself. Partial dependence works by varying the value of the feature under study while the values of all the other features are kept constant. This is a univariate method and thus does not account for potential interactions between covariates, especially when pairs of strongly correlated features exist.

Typically, PDP displays average results, which can compensate for opposite behaviors from different observations. Additionally, altering the values of a single feature while keeping the others constant can create unrealistic feature combinations that do not exist in the actual population. Despite these limitations, PDP is an intuitive tool for visually assessing how relevant factors affect T2D development. For example, in our study, it helps to visually compare how relevant features affect T2D patients versus healthy individuals, enabling the detection of disparities or uneven feature effects.

We used PDPs to provide insights into the features’ contributions and their effects on the predicted outcomes in our prognosis models. By this means, we aim to improve our understanding of the model’s decision-making process.

## Results

This section presents the results obtained. Firstly, we focus on feature selection (via feature importance and backward feature selection). Following this, we describe the models’ performances and the feature partial dependence plots. To conclude, we use the FINDRISC tool as a baseline and compare our models with its results.

### Feature importance

**Fig. 5:**
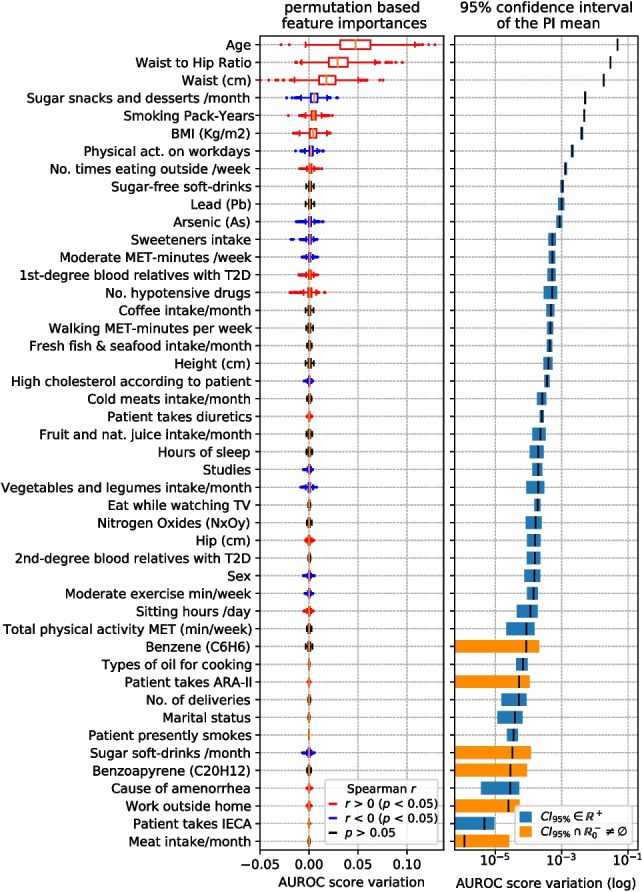
Left: top PI features in the Diagnosis-Environmental (D-ENV) scenario, sorted by importance. Boxplot colors indicate the sign of Spearman’s *r* if *p*-value $<0.05$. Right: 95% CI of mean PI, marking in blue those strictly in $\mathbb {R}^+$

**Fig. 6:**
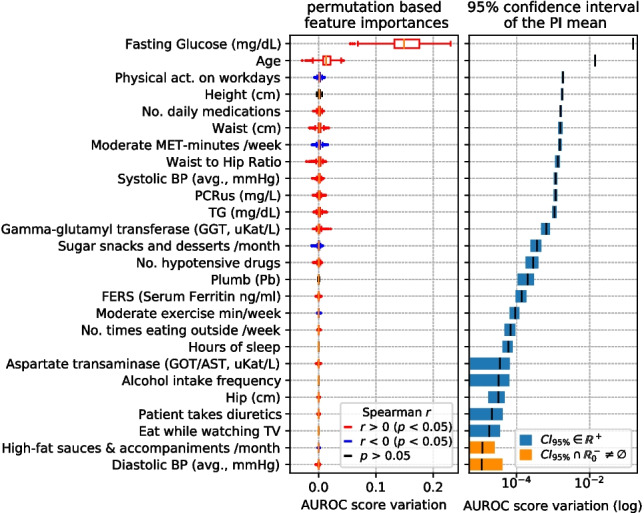
Left: top PI features in the Diagnosis-Healthcare (D-HEA) scenario, sorted by importance. Boxplot colors indicate the sign of Spearman’s *r* if *p*-value $<0.05$. Right: 95% CI of mean PI, marking in blue those strictly in $\mathbb {R}^+$

**Fig. 7:**
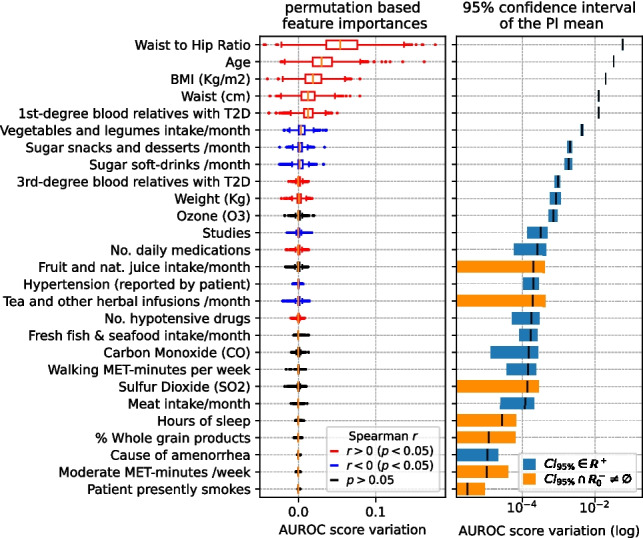
Left: top PI features in the Prognosis-Environmental (P-ENV) scenario, sorted by importance. Boxplot colors indicate the sign of Spearman’s *r* if *p*-value $<0.05$. Right: 95% CI of mean PI, marking in blue those strictly in $\mathbb {R}^+$

**Fig. 8:**
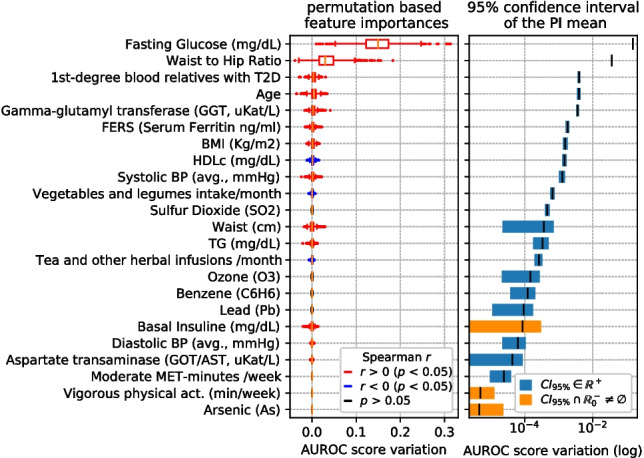
Left: top PI features in the Prognosis-Healthcare (P-HEA) scenario, sorted by importance. Boxplot colors indicate the sign of Spearman’s *r* if *p*-value $<0.05$. Right: 95% CI of mean PI, marking in blue those strictly in $\mathbb {R}^+$

Figures 5, 6, 7, and 8 display, for each scenario, the feature importance ranks obtained using variable permutation importance (PI), in terms of AUROC score variation averaged over 100 repetitions. For each figure, the box and whisker plots are presented on the left, showing the distribution of AUROC score variations for the features. On the right, the mean and the 95% confidence interval (CI) of these variations are shown.

Those features whose AUROC score variation confidence interval of the mean importance does not overlap zero and negative numbers ($\mathbb {R}^-_0$ space) are marked in blue. Conversely, the features whose confidence interval overlaps with $\mathbb {R}^-_0$ (meaning that we cannot ascertain whether they significantly contribute to the model) are marked in orange.

The Spearman’s correlation coefficient (*r*) [46] is computed to individually assess the direction of the relation between each of the shown features and the target, also obtaining the *p*-value of the test. Permutation importance boxplots corresponding to variables with a *p*-value less than 0.05 are colored based on the sign of *r* (blue for negative and red for positive correlation with T2D presence).

### Feature selection

Tables 5, 6, 7, and 8 show the final selected features for the diagnosis and prognosis models, respectively, ordered by mean permutation importance. As can be noticed, the number of features is reduced compared with the initial subset, indicating that not all the most important features are needed to achieve optimal prediction performance. The tables include information about the domain, average value, standard deviation, and range of the features (5–95% quantiles).

**Table 5:** Features selected to conform the T2D Diagnosis-Environmental (D-ENV) model

Feature	Domain	Also in	$\bar{x}$	$S_x$	Q5-Q95
Age	ENV	D-HEA, P-ENV, P-HEA	48.9	16.7	23.0–78.0
Waist to hip ratio	ENV	P-ENV, P-HEA	0.9	0.1	0.7–1.0
Sugar snacks and desserts /month	ENV	P-ENV	65.6	42.3	6.0–150.0
Smoking pack-years	ENV		10.3	19.0	0.0–48.0
BMI (Kg/m2)	ENV	P-ENV	27.8	5.1	20.7–36.8
Sugar-free soft-drinks	ENV	P-ENV	3.1	10.5	0.0–20.0
Arsenic (As)	ENV		1.2	0.8	0.3–2.7
Coffee intake/month	ENV		42.0	34.5	0.0–120.0

**Table 6:** Features selected to conform the T2D Diagnosis-Healthcare (D-HEA) model

Feature	Domain	Also in	$\bar{x}$	$S_x$	Q5-Q95
Fasting glucose (mg/dL)	CLI	P-HEA	94.0	19.5	71.7–118.8
Age	ENV	D-ENV, P-ENV, P-HEA	48.9	16.7	23.0–78.0
Physical act. on workdays	ENV		1.8	0.7	1.0–3.0
Height (cm)	ENV		163.2	9.4	149.0–179.0
Waist (cm)	ENV		92.8	13.9	71.0–116.0
Moderate MET-minutes /week	ENV		559.1	1300.2	0.0–2880.0
No. hypotensive drugs	ENV		0.3	0.6	0.0–2.0
Hours of sleep	ENV		7.3	1.3	5.0–9.3
Aspartate transaminase	CLI	P-HEA	0.3	0.2	0.2–0.5
Hip (cm)	ENV		104.4	9.5	91.0–122.0
Triglyceride (mg/dL)	CLI		117.2	84.5	49.5–234.5

**Table 7:** Features selected to conform the T2D Prognosis-Environmental (P-ENV) model

Feature	Domain	Also in	$\bar{x}$	$S_x$	Q5-Q95
Waist to hip ratio	ENV	D-ENV, P-HEA	0.9	0.1	0.7–1.0
Age	ENV	D-ENV, D-HEA, P-HEA	49.1	14.3	26.0–73.0
BMI (Kg/m2)	ENV	D-ENV	28.0	4.8	21.1–36.8
1st-degree blood relatives with T2D	ENV	P-HEA	0.4	0.6	0.0–1.0
Vegetables and legumes intake/month	ENV	P-HEA	39.5	19.1	13.0–70.0
Sugar snacks and desserts /month	ENV	D-ENV	64.8	41.4	4.0–150.0
Sugar soft-drinks /month	ENV	D-ENV	7.8	14.8	0.0–30.0
Ozone (O3)	ENV		51.1	9.6	34.8–63.8
Fresh fish & seafood intake/month	ENV		10.0	7.1	2.0–20.0
Walking MET-minutes per week	ENV		1348.3	1891.5	0.0–5544.0
Meat intake/month	ENV		16.8	10.2	4.0–30.0

**Table 8:** Features selected to conform the T2D Prognosis-Healthcare (P-HEA) model

Feature	Domain	Also in	$\bar{x}$	$S_x$	Q5-Q95
Fasting glucose (mg/dL)	CLI	D-HEA	94.4	18.5	72.6–117.5
Waist to hip ratio	ENV	D-ENV, P-ENV	0.9	0.1	0.7–1.0
1st-degree blood relatives with T2D	ENV	P-ENV	0.4	0.6	0.0–1.0
Age	ENV	D-ENV, D-HEA, P-ENV	49.1	14.3	26.0–73.0
Gamma-glutamyl transferase	CLI		0.5	0.6	0.2–1.4
HDLc (mg/dL)	CLI		52.8	12.9	33.6–76.1
Vegetables and legumes intake/month	ENV	P-ENV	39.5	19.1	13.0–70.0
Aspartate transaminase	CLI	D-HEA	0.3	0.1	0.2–0.5

**Fig. 9:**
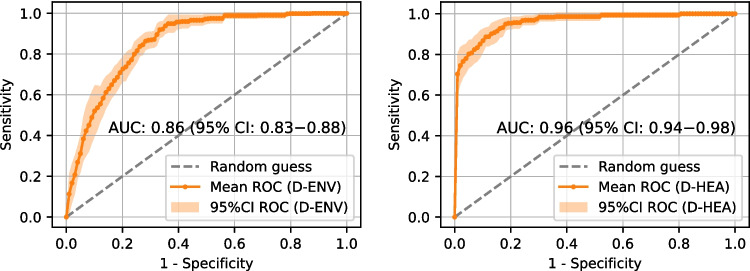
10-fold cross-validation averaged ROC curves obtained from the Diagnosis-Environmental (D-ENV, left) and Diagnosis-Healthcare (D-HEA, right) models

**Fig. 10:**
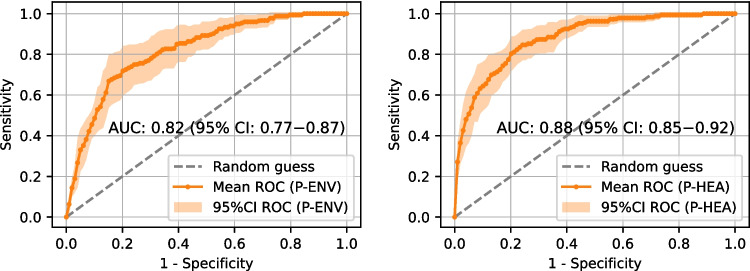
10-fold cross-validation averaged ROC curves obtained from the Prognosis-Environmental (P-ENV, left) and Prognosis-Healthcare (P-HEA, right) models

**Table 9:** Mean ± standard deviation values of test metrics (10-Fold) when setting the decision thresholds at the cutoff points that maximize Youden’s Index (J)

Model	TPR	TNR	PPV	F1-Score	J
D-ENV	0.87±0.07	0.72±0.07	0.16±0.07	0.27±0.09	0.59±0.05
D-HEA	0.86±0.05	0.90±0.06	0.46±0.28	0.56±0.19	0.76±0.06
P-ENV	0.78±0.09	0.75±0.13	0.30±0.09	0.42±0.09	0.52±0.11
P-HEA	0.79±0.08	0.81±0.10	0.37±0.11	0.49±0.09	0.60±0.08

### Model performances

Before the model training process, a hyperparameter optimization was conducted, and the optimized hyperparameters for the models corresponding to each scenario can be found in Supplementary Material 1, Table S13. Following this, models are trained and evaluated in a 10-fold cross-validation. To assess and compare model performances, we calculated their average ROC curves and AUROC values over the 10-fold cross-validation results, as well as performance metrics at the decision thresholds that maximize the Youden’s Index (J) [47].

Figures 9 and 10 display the ROC curves and AUROC values obtained for T2D diagnosis and prognosis models, respectively. Solid curves represent the ROC mean, while the 95% confidence interval of the mean is depicted as a shaded area around them.

Additionally, Table 9 shows, for each of the four scenarios, the true positive rate (TPR, recall, or sensibility), true negative rate (TNR or specificity), positive predictive value (PPV or precision), F1-Score and Youden’s Index (J) when the classification threshold is set to maximize J. The cutoff point that maximizes J, which is the difference between true positive rate (TPR) and false positive rate (1 - TNR), is the point that maximizes the sum of TPR and TNR, thus providing a balanced approach to classification. It is important to consider that, while J gives equal weight to TPR and TNR, misclassification errors often have different associated costs. Therefore, the decision threshold should be tailored to the specific needs and characteristics of the actual real-world scenario.

### Feature partial dependence

**Fig. 11:**
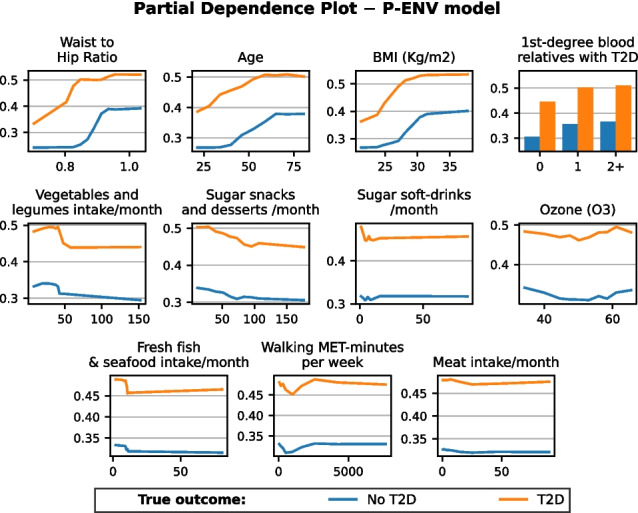
Partial dependence plots (PDP) reflecting univariate feature contribution to T2D development according to the Prognosis-Environmental (P-ENV) model. The *Y*-axis represents the partial dependence, or average expected target response, while the *X*-axis displays the values of each corresponding feature

**Fig. 12:**
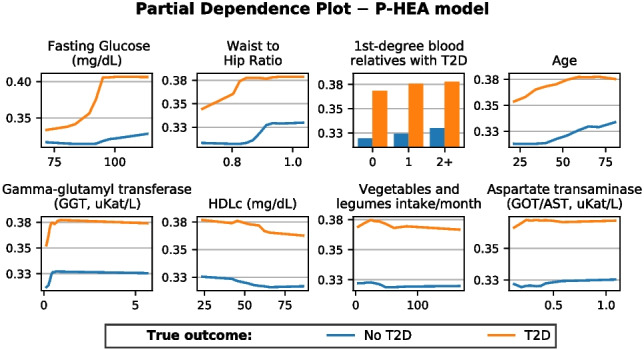
Partial dependence plots (PDP) reflecting univariate feature contribution to T2D development according to the Prognosis-Healthcare (P-HEA) model. The *Y*-axis represents the partial dependence, or average expected target response, while the *X*-axis displays the values of each corresponding feature

Figures 11 and 12 show the contribution to T2D development of the features used to build the two prognosis models, grouping individuals by their diagnosed T2D status.

### FINDRISC comparison

Comparison with other models and solutions presented in other studies is challenging due to various factors, such as differences in cohort sizes, populations, follow-up periods, feature spaces, and other methodology variables. Despite these challenges, we found that FINDRISC, a widely recognized tool with a purpose similar to our proposed models, has sufficient literature with comparable results, making it a feasible benchmark for evaluating our models.

For these reasons, a comparison using the FINDRISC tool was performed, and the results are outlined in Table 10. For reference, we have included the results presented in [19] since the cohort used in this study is also from Spain. Since it was feasible to extract the information needed to fill in the eight questions of the FINDRISC questionnaire from our dataset, we also applied FINDRISC on our own dataset. This way, we can compare the results of FINDRISC and our proposed solutions using the same dataset. Due to the specific features required to compute FINDRISC, the results can only be compared with our D-ENV and P-ENV models. Comparing FINDRISC with the D-HEA and P-HEA models would not be appropriate, as FINDRISC relies on a subset of the features included in the HEA scenario, and the FINDRISC questionnaire does not call for clinical tests to be carried out, which are a component of the HEA models.

**Table 10:** Performance comparison with FINDRISC

Study	Diagnosis	Prognosis
FINDRISC in Spanishpopulation [19]	0.74	0.75
FINDRISC withDi@bet.es data	0.76	0.77
This work(D-ENV, P-ENV)	0.86	0.82

All scores in AUROC

### Test application

The prognosis models (P-ENV and P-HEA) described in this article are available for trial at https://dt2prognosis.iti.es. It is a calculator of the risk of developing T2D in the following 7.5 years, in which the user sends a patient’s data to the model via a form. To enhance user-friendliness, input variables are grouped according to the model in which they are used. After inference, the tool returns the patient’s risk percentile relative to the training population. It should be noted that the tool is available for research purposes only and it has not been validated as a clinical decision tool. Performance and limitations presented in this paper also apply, since the underlying models are the same. Readers wishing to try it should request a username and password by contacting t2d-team@iti.es.

## Discussion

In this work, we analyzed environmental and clinical markers in order to identify factors that may indicate the presence of T2D at a specific moment and to predict T2D development after 7.5 years. We analyzed these data from the general population, including only those who have diabetes but do not know it at the moment of the study, as well as people without diabetes. Moreover, we assessed the presence of T2D after 7.5 years in a subgroup of the sample. We performed geospatial data extraction, missing data imputation, feature engineering, and quasi-constancy filtering processes over the raw Di@bet.es data, merged with information from external geospatial databases.

The feature importance computation identified several informative features associated with T2D presence. As expected, *age* emerged as one of the most prominent factors, consistently scoring highly across all four models. This underscores the critical influence of age on T2D development, a finding supported by previous studies, which have shown its effect becomes particularly evident after 40 years of age [48]. Anthropometric factors, such as *waist circumference*, *waist-to-hip ratio*, and *BMI*, were also consistently relevant in all models, as expected due to the important influence of these factors in T2D development. Among these, *waist-to-hip ratio* played one of the most prominent roles [48–50]. Additionally, the results underscore the importance of some lifestyle factors, such as physical activity and eating habits (*vegetables and legumes*, *fish*, *meat*, and *high-sugar snacks and drinks* intakes), which align with previous findings [51–53]. Note that features can become non-selected because they are either non-informative or redundant, i.e., their information can be statistically inferred from other selected features. This could be the case of *carbohydrate-rich food ingestion*, partially related to *BMI* or *waist-to-hip ratio*.

Family T2D history also played a relevant role in our results, overall in Environmental scenarios where glucose is not considered, and likely due to its association with family history risk factors [54]. *Hours of sleep*, although having been described as an important risk factor [55], was only selected in D-HEA. In addition, factors related to blood pressure (*systolic or diastolic BP*, *hypertension*, or *hypotensive treatments*) are significant across all scenarios, highlighting the association between blood pressure alterations and T2D, both before and alongside its development [56]. The importance of the geospatial information, and derived associated information as income and contamination, reveals the importance of contaminants while the importance of income is not detectable. This can be due to the importance of other factors closely related to income as can be height, BMI, HW ratio and other variables that can mask the effect of this important variable related to T2D development from childhood to adult population [57, 58]. In contrast, contaminants (*lead*, *ozone*, and *arsenic*) appear to be relevant overall in the D-ENV scenario, despite being present in other scenarios. Contaminant markers have been associated with T2D, and our findings could show the importance of air pollution and the limitations in the methodology or data used for assigning contaminant exposure to individuals [23]. In addition, contaminants can be more present in whole population of a city and have lower relationships with economical income in Europe, overall, in Spain [59–61]. In addition, the finetuning of geospatial information can be relevant for improving the results related to it. Overall, these data reinforce the importance of contamination in cardiometabolic disease development [23].

Cigarette smoking is a well-known risk factor for T2D [62]. In our study, we included two smoking-related features. The PI procedure identified *Smoking pack-years* as important in the D-ENV, but *Patient presently smokes* was rejected because of its non-reliable CI. The absence of *Smoking pack-years* in some models could be due to the nature of the feature selection process (backward SFS), meant to find the minimal set of variables that best predicts the target variable (other variables supplied the discriminative power covered by *Smoking pack-years*).

In the Biochemistry or Clinical domain, *fasting glucose* has been identified as the most critical risk factor, as could be expected. In diagnosis, it is a primary diagnostic criterion, and in prediction, elevated glucose levels have been identified as one of the main predictors for T2D development over time [17, 63]. Additionally, *systolic BP*, Gamma-glutamyl transferase (*GGT*), Serum Ferritin (*FERS*), *triglycerides*, and aspartate transaminase *(AST)* have demonstrated statistical relevance in both diagnosis and prognosis. *HDLc*, *fasting insulin*, and *PCRus* were identified as relevant only in the prognosis task.

Regarding model performances, the number of features selected by the SFS algorithm is relatively small, ranging from 8 to 11, depending on the model. Age is the only feature included in all four models, while the *waist-to-hip ratio* appears in three of them (although waist and hip measurements are present in the remaining model). In P-ENV, the selected features include key known risk factors such as *waist-to-hip ratio*, *age*, *BMI*, *first-degree relatives*, as well as other factors that reduce the risk, such as *vegetables and legume intake*, *fresh fish and seafood intake*, and *meat intake*. While the effect of monthly sugar snacks and desserts and sugar-sweetened soft-drinks seems contradictory, it is important to note that these foods constitute only a small portion of total food consumption in our population. In addition, exercise (*walking MET-minutes per week*) indicates that reduced levels of exercise increase the risk, while high levels of exercise have a limited effect.

Regarding the features selected by group, when available, CLI features are present to a greater extent in the models, especially in the prognosis model (P-HEA), where four out of eight were selected (*fasting glucose*, *GGT*, *HDLc*, and *AST*), despite CLI features being only a 16% of the total. Fasting glucose levels have been traditionally identified as an important predictive marker for T2D [17], along with *GGT*, *HDL*, *GGT/ratio* and *AST* [64–66], in spite of some disagreement between authors [67, 68]. Furthermore, high levels of *GGT* and *AST*, along with low levels of *HDL*, are relevant biomarkers of non-alcoholic fatty liver disease (NAFLD), which is also related to T2D development [69]. These findings underscore the importance of age, glucose levels, body composition, and liver alterations as relevant predictors of incident T2D in the population. In this P-HEA model, the features from the Environmental domain (*Age*, *Waist-to-hip ratio*, *blood relatives*, and *vegetable intake*) were also selected for the P-ENV model.

The P-HEA model’s partial dependence plots (PDPs) show that the risk increases drastically when *fasting glucose* exceeds 90 mg/dL for T2D patients, as previously reported [17]. Similar to the P-ENV scenario, *waist-to-hip ratio*, *age*, *GGT*, and having relatives with T2D are associated with an increased risk of developing T2D. Higher GGT values also appear to increase the risk, consistent with previous studies [70, 71]. However, it remains unclear whether this increase is directly associated with T2D or with other related risk factors (such as obesity or NAFLD) [72]. It is also noteworthy that low HDLc levels appear to increase the risk. Although this has been observed as a risk factor, authors in [73] concluded that this association might be influenced by confounding factors. According to [74], individuals with high HDLc levels tend to have lower body mass index, waist circumference, and glucose levels, i.e., lower risk for T2D.

Diagnosis using environmental factors alone achieves an AUROC of 0.86 (95% CI: 0.83−0.88). However, from the perspective of the unbalanced T2D problem, this model shows the lowest F1-Score (0.29) among all the tested models, which suggests that it is not a good choice for diagnosing T2D using only environmental features. This improves to 0.96 (95% CI: 0.94−0.98) when features from the clinical domain are added. Although incorporating biochemistry analysis features may increase costs, the benefits are substantial. A similar trend is observed in the prognosis task (trained with a 7-year follow-up), where the AUROC values are 0.82 (95% CI: 0.77−0.87) with environmental factors alone, and 0.88 (95% CI: 0.85−0.92) when clinical features are included. These results suggest that these models could effectively support clinicians in diagnosis and prognosis tasks using readily accessible patient data with high confidence.

It is worth noting that performance in the HEA scenario is superior to that in the ENV scenario. This difference is primarily due to the variables included in each case. Specifically, the features in the ENV scenario are a subset of those in the HEA scenario. The latter includes variables such as *fasting glucose*, which is a well-known factor in T2D development as mentioned before. Moreover, environmental features include self-reported data, which may be less precise compared to biochemistry measurements, potentially diminishing performance, especially in the Environmental scenario. Furthermore, interactions between features of the two different feature domains can only be considered by the models in the HEA scenario, which likely benefits their performance given the complexity of T2D.

Both our Environmental diagnosis and prognosis results surpass those obtained with FINDRISC in [19] ($0.86 > 0.74$ AUROC when diagnosing unknown T2D, $0.82 > 0.75$ AUROC in predicting incident T2D). This study was also conducted in Spain; however, there are significant differences in cohort size and follow-up time. For reference and fair performance comparison, our AUROC scores using the FINDRISC survey on our own dataset are 0.775 for diagnosis and 0.766 for prognosis. These scores are slightly higher but close to those reported by [19], and lower than the machine learning model scores we achieved using our own custom feature subsets. This indicates that, despite the similarities in task difficulty between the cohorts, our proposed methodology outperforms FINDRISC in both diagnosis and prognosis.

### Limitations

It should be noted that some variables, particularly lifestyle-related ones such as smoking frequency, coffee intake, and nutrition, are collected via patient’s self-reported surveys. These self-reported variables may lack the precision of other measurements. Furthermore, the dataset does not include variables related to psychological stress (a risk factor found in the literature [75]). These issues could impact model performance.

Missing values have been found to various degrees across different features, forming distinct patterns of missingness. For example, in Fig. 3, it appears that some patients have numerous features related to diet or geospatial information missing, while others have all this data complete. Although this could lead to introducing bias in imputations, we have found that none of the most important variables in the four models exhibit a significant amount of missing values. Therefore, we can reasonably assume that the most informative features were not deemed relevant due to an overfit to bias introduced by imputation, although some degree of impact on other variables cannot be ruled out.

While this work presents some model explainability through partial dependence plots (PDPs), XGboost is a complex model that is not directly interpretable. Furthermore, neither PDPs nor other explainability techniques are available in the tool presented in Section 3.6. This lack of explainability could hinder the adoption of these models in clinical practice.

Lastly, the models are only evaluated using the Di@bet.es study. Nonetheless, the models could be evaluated on other datasets that share variables and units. Results should be comparable if the population is similar, which could be evaluated via statistical tests such as the Hotelling test [76]. Otherwise, the same methodology could be used to create new models for this specific population.

## Conclusions

This work presents a comprehensive machine learning-oriented study on T2D diagnosis and prognosis using the Di@betes.es dataset. After improving data quality, we applied feature importance and sequential feature selection (SFS) techniques to identify and list the most predictive features across four scenarios (scenario-task pairs). These feature subsets were used to train four models, achieving 0.86, 0.96, 0.82 and 0.88 AUROC in the D-ENV, D-HEA, P-ENV, and P-HEA models, respectively. Combining biochemistry features with environmental ones yielded better performance, although this may involve higher costs. The prognosis models (P-ENV and P-HEA) described in this article are available for trial at https://dt2prognosis.iti.es upon request.

The identification of reduced and optimal feature subsets for each scenario has significant practical implications. It facilitates the use of simpler screening surveys and clinical tests to estimate the risk of developing T2D, thereby enabling more efficient resource allocation. By pinpointing patients with a higher risk of developing the disease, healthcare providers can prioritize more accurate and detailed tests for those individuals.

Genetic information, which is not included in this work, could potentially enhance performance, particularly for individuals with no well-known risk factors. Additionally, more complex models, such as deep-learning models, could be explored to improve performance, although they may compromise explainability. On the other hand, using permutation importance for feature selection might only capture some complex interactions. Exploring other feature selection techniques could be a valuable future improvement to consider. Lastly, as we have stated in our Limitations, the lack of model explainability could hinder its adoption. Therefore, future work could focus on integrating other well-established methods for local explainability, such as SHAP [77] or LIME [78].

## Supplementary Information

Below is the link to the electronic supplementary material.

Supplementary file 1 (pdf 222 KB)

## Data Availability

The data that supports the findings of this study are not openly available due to reasons of sensitivity. The authors do not have the authority to disclose these data, but they are available upon reasonable request by contacting the corresponding authors of “Prevalence of diabetes mellitus and impaired glucose regulation in Spain: the Di@bet.es Study, Diabetologia, 2012” (10.1007/s00125-011-2336-9).

## Footnotes

1 Details on the software used in this study can be found in Supplementary Material 1, Section 4.

2 This measure, defined by the INE (National Statistics Institute, Spain), is calculated by dividing total household income by the number of consumption units.

3 Regions were identified with Spanish *Comunidades Autónomas*.
