# Supplementary material for: A machine learning approach for type 2 diabetes diagnosis and prognosis using tailored heterogeneous feature subsets
Source: Med Biol Eng Comput. 2025 Apr 8;63(9):2733–52. doi: 10.1007/s11517-025-03355-5 (PMC12402034; doi:10.1007/s11517-025-03355-5)
Supplement: Supplementary file 1 — (pdf 222 KB) [file 11517_2025_3355_MOESM1_ESM.pdf]

A Machine Learning Approach for Type 2 Diabetes Diagnosis and Prognosis Using Tailored Heterogeneous Feature Subsets

S1 Appendix: Details on Dataset Features, Algorithms, and Models

J.Ramón Navarro-Cerdán[1,2,*], Pedro Pons-Suñer[2], Laura Arnal[2], Joaquim Arlandis[1,2], Rafael Llobet[1,2], Juan-Carlos Perez-Cortes[1,2], Francisco Lara-Hernández[3], Celeste Moya-Valera[3], Maria Elena Quiroz-Rodriguez[3], Gemma Rojo-Martinez[4,5], Sergio Valdés[4,5], Eduard Montanya[4,6,7], Alfonso L. Calle-Pascual[8,9], Josep Franch-Nadal[4,10], Elias Delgado[11,12], Luis Castaño[4,12,13], Ana-Bárbara García-García[3,4], Felipe Javier Chaves[3,4]

[*] Corresponding author. e-mail: jonacer@upv.es
[1]Universitat Politècnica de València, Camí de Vera, s/n, València, 46022, Spain
[2] ITI, Universitat Politècnica de València, Camino de Vera s/n València, 46022, Spain
[3] Genomic and Diabetes Unit, INCLIVA Biomedical Research Institute, 46010, Spain
[4] CIBERDEM, ISCIII, Madrid, Spain
[5] UGC Endocrinología y Nutrición, Hospital regional Universitario de Málaga, Instituto de Investigación Biomédica de Málaga y Plataforma en Nanomedicina-IBIMA Plataforma BIONAND, Málaga, Spain
[6] Bellvitge Hospital-IDIBELL, Barcelona, Spain
[7] Department of Clinical Sciences, Barcelona, Spain
[8] Medical School, University Complutense, Madrid, Spain
[9] Endocrinology and Nutrition Department, Hospital Clínico Universitario San Carlos, Madrid, Spain
[10] EAP Raval Sud, Catalan Institute of Health, GEDAPS Network, Primary Care, Research Support Unit (IDIAP-Jordi Gol Foundation, Barcelona, Spain
[11] Department of Endocrinology and Nutrition, Central University Hospital of Asturias/University of Oviedo, Health Research Institute of the Principality of Asturias (ISPA), Oviedo, Spain
[12] CIBERER, Madrid, Spain
[13] Cruces University Hospital, Biocruces Bizkaia Health Research Institute, Endo-ERN, UPV/EHU, Barakaldo, Spain

1 Detailed list of dataset features

In this section, features used in this study are displayed in several tables, paired with their respective short descriptions. For convenience, they are shown in three different tables:

- Clinical features (Table S1).
- Environmental geospatial features (Table S2).
- Other environmental features (Table S3).

Table S1. Names and short descriptions of clinical (CLI) features.

Name	Description
Total cholesterol (mg/dL)	Total cholesterol (mg/dL)
Creatinine (umol/L)	Creatinine (umol/L)
FERS (Serum Ferritin ng/ml)	FERS (Serum Ferritin, ng/ml)
Gamma-glutamyl transferase (GGT, uKat/L)	Gamma-glutamyl transferase (GGT, uKat/L)
Fasting Glucose (mg/dL)	Fasting Glucose (mg/dL)
Aspartate transaminase (GOT/AST, uKat/L)	Aspartate transaminase (GOT/AST, uKat/L)
GGPT (uKat/L)	Gamma-glutamyl transpeptidase (GGPT, uKat/L)
HDLc (mg/dL)	High-density lipoprotein cholesterol (HDLc, mg/dL)
HIES (Iron (II+III) in serum) (umol/L)	HIES (Iron (II+III) in serum) (umol/L)
Fasting Insulin (mg/dL)	Fasting Insulin (mg/dL)
LDLc (mg/dL)	Low-density lipoprotein cholesterol (LDLc, mg/dL)
PCRus (mg/L)	Ultra-sensitive C-reactive protein (mg/L)
TG (mg/dL)	Triglycerides (mg/dL)
Serum Uric Acid (umol/L)	Serum Uric Acid (umol/L)
Systolic BP (avg., mmHg)	Average Systolic Blood Pressure (mmHg)
Diastolic BP (avg., mmHg)	Average Diastolic Blood Pressure (mmHg)

Table S2. Names and short descriptions of environmental (ENV) geospatial features.

Name	Description
Nitrogen Oxides (NxOy)	Ambient air pollution: Nitrogen Oxides (NxOy) (μg/m3)
Particle pollution (PM10)	Ambient air pollution: Inhalable Particulate Matter <10μg (μg/m3)
Nitrogen Dioxide (NO2)	Ambient air pollution: Nitrogen Dioxide (NO2) (μg/m3)
Arsenic (As)	Ambient air pollution: Arsenic (As) (ng/m3)
Income per consumption unit	Average income per consumption unit in the municipality.
Nickel (Ni)	Ambient air pollution: Nickel (Ni) (ng/m3)
Nitrogen Monoxide (NO)	Ambient air pollution: Nitrogen Monoxide (NO) (μg/m3)
Benzene (C6H6)	Ambient air pollution: Benzene (C6H6) (μg/m3)
Cadmium (Cd)	Ambient air pollution: Cadmium (Cd) (ng/m3)
Particle pollution (PM2.5)	Ambient air pollution: Inhalable Particulate Matter <2μg (μg/m3)
Ozone (O3)	Ambient air pollution: Ozone (O3) (μg/m3)
Carbon Monoxide (CO)	Ambient air pollution: Carbon Monoxide (CO) (mg/m3)
Population	Number of inhabitants in the municipality.
Benzoapyrene (C20H12)	Ambient air pollution: Benzoapyrene (C20H12) (ng/m3)
Plumb (Pb)	Ambient air pollution: Plumb (Pb) (μg/m3)
Sulfur Dioxide (SO2)	Ambient air pollution: Sulfur Dioxide (SO2) (μg/m3)

Table S3. Names and short descriptions of environmental (ENV) features.

Name	Description
Age	Age of the patient in years.
Sex	Sex of the patient.
Pregnant	Pregnancy status.
Labor < 6 months	Had a childbirth in the last 6 months.
Surgeries	Has had any surgeries.
Severe illness	Has suffered any severe illness.
Marital status	Marital status (single/married/widowed/separated)
Work outside home	Patient works outside the home.
Occupation	Occupation (7 categories ranging from executives to unskilled manual workers)
Patient presently smokes	The patient has indicated that they presently smoke.
Hours of sleep	Total hours of sleep.
Physical act. on workdays	Level of physical activity at work (sit/stand/carry/heavy)
Hypertension (reported by patient)	The patient reports having hypertension.
High cholesterol according to patient	The patient reports having high cholesterol.
Cardiovascular accident	The patient has experienced a cardiovascular accident.
Stroke	The patient has had a stroke.
Peripheral vasculopathy	The patient has peripherial vasculopathy.
Lipid-Lowering Drug Therapy	The patient is undergoing lipid-lowering drug therapy.
Takes thyroid hormones	The patient takes thyroid hormones.
Takes uricosurics	The patient takes uricosurics.
Patient takes psychotropics	The patient takes psychotropics.
Patient takes AINES	The patient takes nonsteroidal anti-inflammatory drugs (NSAIDs/AINEs)
No. daily medications	Number of medications taken daily.
No. of deliveries	Number of childbirths the patient has had.
No. of cases of gestational diabetes	Number of times the patient has had gestational diabetes.
Hormonal contraceptives	The patient takes hormonal contraceptives.
Cause of amenorrhea	If the patient has amenorrhea, is it due to natural or surgical causes?
Menopause treatment	The patient is undergoing treatment for her menopause.
Weight (Kg)	Weight of the patient (Kg)
Height (cm)	Height of the patient (cm)
Waist (cm)	Waist circumference (cm)
Hip (cm)	Hip circumference (cm)
No. times eating outside /week	Number of times the patient eats outside per week.
Vitamin supplements	The patient takes vitamin supplements.
Sweeteners intake	The patient regularly uses saccharin or other no calorie sweeteners.
Fructose intake (y/n)	The patient regularly consumes fructose.
Types of salt for cooking	What type of salt does the patient use for cooking? (sea/iodized/other)
No. of meals /day	How many meals does the patient usually have on a normal day?
Snaks between meals	Does the patient eat snacks between meals? (yes/no/sometimes)
Eat while watching TV	Does the patient watch TV while eating? (yes/no/sometimes)
Fruit and nat. juice intake/month	Fruit and natural juice consumption per month.
Nuts intake/month	Nuts consumption per month.
Fresh fish & seafood intake/month	Fresh fish and seafood consumption per month.
Canned fish & seafood intake/month	Canned fish and seafood consumption per month.
Meat intake/month	Meat consumption per month.
Cold meats intake/month	Cold meats consumption per month.
Alcohol intake frequency	Alcohol intake frequency (daily/occasionally/weekend/never)
Sugar soft-drinks /month	Sugar soft-drinks consumption per month.
Sugar-free soft-drinks	Sugar-free soft-drinks consumption per month.
Packaged juice /month	Packaged juice consumption per month.
Vigorous physical act. (min/week)	Total time spent on vigorous physical activities on active days (min/week)
Moderate exercise min/week	Total time spent on moderate physical activities on active days (min/week)
Walking minutes per week	Total time spent walking on active days (min/week)
Studies	Education level (4 categories ranging from No to University studies)
BMI (Kg/m2)	Body Mass Index (Kg/m2)
Waist to Hip Ratio	Waist (cm) to Hip (cm) Ratio.
Patient takes IECA	The patient takes angiotensin converting enzyme inhibitors.
Patient takes ARA-II	The patient takes angiotensin II receptor antagonists.
Takes beta-blockers	The patient takes beta-blockers.
Calcium channel blockers	The patient takes calcium channel blockers.
Patient takes diuretics	The patient takes diuretics.
Takes alpha-blockers	The patient takes alpha-blockers.
Renin inhibitors	The patient takes renin inhibitors.
No. hypotensive drugs	Number of hypotensive drugs that the patient takes.
Walking MET-minutes per week	Walking MET-minutes per week.
Moderate MET-minutes /week	Moderate physical activity MET-minutes per week.
Vigorous MET-minutes /week	Vigorous physical activity MET-minutes per week.
Total physical activity MET (min/week)	Total physical activity MET (min/week)
Workout hours per week	Total hours per week dedicated to physical exercise.
Sitting hours /day	Total sitting hours per day.
Smoking Pack-Years	Smoking Pack-Years (cigarettes per day * years smoking)
1st-degree blood relatives with T2D	Number of 1st-degree blood relatives with T2D.
2nd-degree blood relatives with T2D	Number of 2nd-degree blood relatives with T2D.
3rd-degree blood relatives with T2D	Number of 3rd-degree blood relatives with T2D.
Tea and other herbal infusions /month	Tea and other herbal infusions consumption per month.
Coffee intake/month	Coffee consumption per month
Beverages with caffeine /month	Caffeinated beverages consumption per month.
Wine intake/month	Wine consumption per month
Vegetables and legumes intake/month	Vegetables and legumes consumption per month.
Fried & precooked food intake/month	Fried and precooked food consumption per month.
Eggs and dairy /month	Eggs and dairy products consumption per month.
Bread, rice, pasta intake/month	Bread, rice, and pasta consumption per month.
% Whole grain products	Percentage of whole grain consumption (pasta, rice, bread)
Sugar snacks and desserts /month	Consumption of sugary snacks and desserts per month.
High-fat sauces & accompaniments /month	Consumption of saucess and accompaniments high in fats per month.
Changes to improve diet	The patient has been advised by a doctor to change their diet.
Beer intake/month	Beer consumption per month.
Types of oil for cooking	Type of oil used for cooking.

2 Missing data imputation of dataset features

In the manuscript, a Quality Score based on missingness of data and imputation scores is presented, calculated following Equation 1.

$$\text{QS} = \gamma + (1 - \gamma) * \mu \tag{1}$$

where:

$$\gamma = \% \text{ of complete entries in the column, [0–1]}$$
$$\mu = \text{imputation score, e.g. } R^2 \text{ score, [0–1]}$$

The imputation method used for filling missing values in each feature is tailored using the IQA algorithm (`https://doi.org/10.48550/arXiv.2407.11767`). An imputer from an available collection of methods is assigned to each feature not only based on their imputation score, but also trying to minimize biases introduced by imputation. Table S4 displays the imputation methods that were analyzed by IQA. The type of imputation method is also indicated (univariate or multivariate). The A Priori Probabilistic Random (APPRandom) Imputer fills missing values drawing random values from the complete entries, preserving the distribution of complete data, and is intended to minimize imputation bias without trying to "guess" the true value.

Table S4. Imputers analyzed by IQA.

**Imputer**	**ID**	**Type**	**Parameters**
Simple Imputer	Mean	Univar.	strategy: mean
Simple Imputer	Median	Univar.	strategy: median
Simple Imputer	Mode	Univar.	strategy: mode
APPRandom	APPRandom	Univar.	—
K-Nearest Neighbors	KNNImputer	Multiv.	n_neighbors: 5
Iterative Imputer	IterativeRF	Multiv.	init_strategy: mode<br>max_iter: 20<br>estimator: RFRegressor(<br>↪ n_estimators: 100<br>)

In this section, features are presented with their respective chosen imputer, the imputation score of said imputers, their completeness, and the quality score obtained with Equation 1. For convenience, they are shown in three different tables:

- Clinical features (Table S5).
- Environmental geospatial features (Table S6).
- Other environmental features (Table S7).

Table S5. Chosen imputer, imputation score of the chosen imputer (μ), completeness (γ), and final quality score (QS) of clinical (CLI) features.

Name	Chosen imputer	μ	γ	QS
Total cholesterol (mg/dL)	IterativeRF	0.93	1.0	1.0
Creatinine (umol/L)	IterativeRF	0.43	1.0	1.0
FERS (Serum Ferritin ng/ml)	IterativeRF	0.22	1.0	1.0
Gamma-glutamyl transferase (GGT, uKat/L)	IterativeRF	0.21	1.0	1.0
Fasting Glucose (mg/dL)	IterativeRF	0.16	1.0	1.0
Aspartate transaminase (GOT/AST, uKat/L)	IterativeRF	0.46	1.0	1.0
GGPT (uKat/L)	IterativeRF	0.57	0.98	0.99
HDLc (mg/dL)	IterativeRF	0.58	1.0	1.0
HIES (Iron (II+III) in serum) (umol/L)	IterativeRF	0.21	1.0	1.0
Fasting Insulin (mg/dL)	IterativeRF	0.11	0.99	0.99
LDLc (mg/dL)	IterativeRF	0.92	1.0	1.0
PCRus (mg/L)	IterativeRF	0.05	0.99	0.99
TG (mg/dL)	IterativeRF	0.36	1.0	1.0
Serum Uric Acid (umol/L)	IterativeRF	0.46	0.99	1.0
Systolic BP (avg., mmHg)	IterativeRF	0.62	1.0	1.0
Diastolic BP (avg., mmHg)	IterativeRF	0.32	1.0	1.0

Table S6. Chosen imputer, imputation score of the chosen imputer (μ), missingness fraction $(1-\gamma/100)$, and final quality score (QS) of geospatial environmental (ENV) features.

Name	Chosen imputer	μ	γ	QS
Nitrogen Oxides (NxOy)	IterativeRF	0.97	1.0	1.0
Particle pollution (PM10)	IterativeRF	0.3	1.0	1.0
Nitrogen Dioxide (NO2)	IterativeRF	0.89	1.0	1.0
Arsenic (As)	IterativeRF	0.51	0.97	0.99
Income per consumption unit	IterativeRF	0.23	1.0	1.0
Nickel (Ni)	IterativeRF	0.16	0.97	0.98
Nitrogen Monoxide (NO)	IterativeRF	0.93	1.0	1.0
Benzene (C6H6)	IterativeRF	0.46	1.0	1.0
Cadmium (Cd)	IterativeRF	0.29	0.97	0.98
Particle pollution (PM2.5)	IterativeRF	0.35	0.84	0.9
Ozone (O3)	IterativeRF	0.47	1.0	1.0
Carbon Monoxide (CO)	IterativeRF	0.19	1.0	1.0
Population	IterativeRF	0.65	1.0	1.0
Benzoapyrene (C20H12)	APPRandom	0.0	0.97	0.97
Plumb (Pb)	IterativeRF	0.06	1.0	1.0
Sulfur Dioxide (SO2)	IterativeRF	0.37	1.0	1.0

Table S7. Chosen imputer, imputation score of the chosen imputer (μ), missingness fraction ($1 - \gamma/100$), and final quality score (QS) of environmental (ENV) features.

Name	Chosen imputer	μ	γ	QS
Age	IterativeRF	0.67	1.0	1.0
Sex	IterativeRF	0.94	1.0	1.0
Pregnant	APPRandom	1.0	1.0	1.0
Labor < 6months	APPRandom	1.0	1.0	1.0
Surgeries	APPRandom	1.0	1.0	1.0
Severe illness	APPRandom	1.0	1.0	1.0
Marital status	IterativeRF	0.28	1.0	1.0
Work outside home	IterativeRF	0.7	1.0	1.0
Occupation	IterativeRF	0.19	1.0	1.0
Patient presently smokes	IterativeRF	0.6	1.0	1.0
Hours of sleep	IterativeRF	0.02	1.0	1.0
Physical act. on workdays	IterativeRF	0.1	1.0	1.0
Hypertension (reported by patient)	IterativeRF	0.62	0.99	1.0
High cholesterol according to patient	IterativeRF	0.35	0.99	0.99
Cardiovascular accident	IterativeRF	0.24	1.0	1.0
Stroke	IterativeRF	0.01	0.99	0.99
Peripheral vasculopathy	Mode	0.0	1.0	1.0
Lipid-Lowering Drug Therapy	IterativeRF	0.53	1.0	1.0
Takes thyroid hormones	Mode	0.01	1.0	1.0
Takes uricosurics	IterativeRF	0.05	1.0	1.0
Patient takes psychotropics	IterativeRF	0.17	1.0	1.0
Patient takes AINES	IterativeRF	0.14	1.0	1.0
No. daily medications	IterativeRF	0.61	1.0	1.0
No. of deliveries	IterativeRF	0.47	1.0	1.0
No. of cases of gestational diabetes	IterativeRF	0.0	1.0	1.0
Hormonal contraceptives	Mode	0.14	1.0	1.0
Cause of amenorrhea	IterativeRF	0.42	1.0	1.0
Menopause treatment	IterativeRF	0.06	0.96	0.96
Weight (Kg)	IterativeRF	0.98	0.99	1.0
Height (cm)	IterativeRF	0.82	0.99	1.0
Waist (cm)	IterativeRF	0.97	0.99	1.0
Hip (cm)	IterativeRF	0.82	0.99	1.0
No. times eating outside /week	IterativeRF	0.15	1.0	1.0
Vitamin supplements	APPRandom	0.0	0.99	0.99
Sweeteners intake	IterativeRF	0.15	1.0	1.0
Fructose intake (y/n)	KNNImputer	0.03	0.99	0.99
Types of salt for cooking	IterativeRF	0.11	0.86	0.88
No. of meals /day	APPRandom	0.0	1.0	1.0
Snaks between meals	APPRandom	0.0	1.0	1.0
Eat while watching TV	Mode	0.5	0.93	0.96
Fruit and nat. juice intake/month	IterativeRF	0.07	0.89	0.9
Nuts intake/month	IterativeRF	0.01	0.89	0.89
Fresh fish & seafood intake/month	IterativeRF	0.08	0.89	0.9
Canned fish & seafood intake/month	IterativeRF	0.03	0.89	0.89
Meat intake/month	IterativeRF	0.01	0.89	0.89
Cold meats intake/month	IterativeRF	0.01	0.89	0.89
Alcohol intake frequency	IterativeRF	0.71	0.71	0.92
Sugar soft-drinks /month	IterativeRF	0.21	0.89	0.91
Sugar-free soft-drinks	IterativeRF	0.01	0.89	0.89
Packaged juice /month	IterativeRF	0.02	0.89	0.89
Vigorous physical act. (min/week)	IterativeRF	0.54	0.66	0.84
Moderate exercise min/week	IterativeRF	0.41	0.74	0.85
Walking minutes per week	IterativeRF	0.24	0.97	0.97
Studies	IterativeRF	0.27	1.0	1.0
BMI (Kg/m2)	IterativeRF	0.91	0.99	1.0
Waist to Hip Ratio	IterativeRF	0.97	0.99	1.0
Patient takes IECA	IterativeRF	0.65	1.0	1.0
Patient takes ARA-II	IterativeRF	0.68	1.0	1.0
Takes beta-blockers	IterativeRF	0.45	1.0	1.0
Calcium channel blockers	IterativeRF	0.54	1.0	1.0
Patient takes diuretics	IterativeRF	0.68	1.0	1.0
Takes alpha-blockers	IterativeRF	0.24	1.0	1.0
Renin inhibitors	Mode	0.0	1.0	1.0
No. hypotensive drugs	IterativeRF	0.91	1.0	1.0
Walking MET-minutes per week	IterativeRF	0.89	0.99	1.0
Moderate MET-minutes /week	IterativeRF	0.69	0.99	1.0
Vigorous MET-minutes /week	IterativeRF	0.51	0.99	1.0
Total physical activity MET (min/week)	IterativeRF	0.94	0.99	1.0
Workout hours per week	KNNImputer	0.03	1.0	1.0
Sitting hours /day	IterativeRF	0.34	0.99	1.0
Smoking Pack-Years	IterativeRF	0.29	0.99	1.0
1st-degree blood relatives with T2D	APPRandom	0.0	1.0	1.0
2nd-degree blood relatives with T2D	APPRandom	0.0	1.0	1.0
3rd-degree blood relatives with T2D	APPRandom	0.0	1.0	1.0
Tea and other herbal infusions /month	IterativeRF	0.0	0.89	0.89
Coffee intake/month	IterativeRF	0.64	0.89	0.96
Beverages with caffeine /month	IterativeRF	0.63	0.89	0.96
Wine intake/month	IterativeRF	0.36	0.89	0.93
Vegetables and legumes intake/month	IterativeRF	0.12	0.89	0.9
Fried & precooked food intake/month	IterativeRF	0.12	0.89	0.9
Eggs and dairy /month	IterativeRF	0.05	0.89	0.9
Bread, rice, pasta intake/month	IterativeRF	0.05	0.89	0.9
% Whole grain products	IterativeRF	0.11	0.89	0.9
Sugar snacks and desserts /month	IterativeRF	0.19	0.89	0.91
High-fat sauces & accompaniments /month	IterativeRF	0.04	0.89	0.89
Changes to improve diet	IterativeRF	0.21	1.0	1.0
Beer intake/month	IterativeRF	0.22	0.89	0.91
Types of oil for cooking	KNNImputer	0.01	0.89	0.89

3 Quasi-constancy filtering

In our manuscript, we detail the process of filtering features based on various quality criteria. One of these criteria is feature constancy, evaluated using a novel quasi-constancy index derived from the Gini coefficient of inequality, denoted as $\mathcal{G}$. A threshold of 0.05 is established for this index, determined through empirical analysis of thousands of simulations. This section provides a more comprehensive explanation of the empirical process used to derive this threshold.

Smaller datasets are particularly susceptible to the effects of quasi-constant variables, as models struggle to learn patterns from groups of observations that deviate from the mode when these groups are too small. Conceptually, the recommended threshold for a quasi-constancy filter should depend on the dataset size. Through thousands of synthetic experiments with random sample sizes, we observed that this relationship follows a negative exponential curve, expressed as:

$$f(x) = a * \exp(-b * x) + c, \text{ where x is the dataset size}$$

We measured the minimum quasi-constancy coefficient (QCC) of variables that had an impact on the target, based on non-negative permutation importances (with a ±95% confidence interval). Setting the threshold above these QCC values would have resulted in the removal of at least one important variable. By fitting the curve that relates sample size to QCC, we empirically determined the coefficients to be:

- $a = 0.169$
- $b = 0.016$
- $c = 0.05$

Thus, for our dataset's sample size, a QCC threshold of 0.05 or lower is recommended. Features along with their QCC are listed in Tables S8, S9, and S10, grouped by domain.

Table S8. Names and quasi-constancy coefficient of clinical (CLI) features.

Name	Quasi-Constancy Coefficient
Total cholesterol (mg/dL)	0.13
Creatinine (umol/L)	0.12
FERS (Serum Ferritin ng/ml)	0.18
Gamma-glutamyl transferase (GGT, uKat/L)	0.11
Fasting Glucose (mg/dL)	0.1
Aspartate transaminase (GOT/AST, uKat/L)	0.13
GGPT (uKat/L)	0.2
HDLc (mg/dL)	0.13
HIES (Iron (II+III) in serum) (umol/L)	0.18
Fasting Insulin (mg/dL)	0.15
LDLc (mg/dL)	0.16
PCRus (mg/L)	0.15
TG (mg/dL)	0.15
Serum Uric Acid (umol/L)	0.13
Systolic BP (avg., mmHg)	0.18
Diastolic BP (avg., mmHg)	0.13

Table S9. Names and quasi-constancy coefficient of environmental (ENV) geospatial features.

Name	Quasi-Constancy Coefficient
Nitrogen Oxides (NxOy)	0.2
Particle pollution (PM10)	0.18
Nitrogen Dioxide (NO2)	0.19
Arsenic (As)	0.2
Income per consumption unit	0.11
Nickel (Ni)	0.18
Nitrogen Monoxide (NO)	0.21
Benzene (C6H6)	0.2
Cadmium (Cd)	0.22
Particle pollution (PM2.5)	0.17
Ozone (O3)	0.22
Carbon Monoxide (CO)	0.21
Population	0.13
Benzoapyrene (C20H12)	0.22
Plumb (Pb)	0.05
Sulfur Dioxide (SO2)	0.22

Table S10. Names and quasi-constancy coefficient of environmental (ENV) features.

Name	Quasi-Constancy Coefficient
Age	0.22
Sex	0.23
Pregnant	0.0
Labor < 6months	0.0
Surgeries	0.0
Severe illness	0.0
Marital status	0.18
Work outside home	0.25
Occupation	0.24
Patient presently smokes	0.17
Hours of sleep	0.14
Physical act. on workdays	0.26
Hypertension (reported by patient)	0.16
High cholesterol according to patient	0.19
Cardiovascular accident	0.03
Stroke	0.02
Peripheral vasculopathy	0.0
Lipid-Lowering Drug Therapy	0.08
Takes thyroid hormones	0.03
Takes uricosurics	0.01
Patient takes psychotropics	0.1
Patient takes AINES	0.09
No. daily medications	0.21
No. of deliveries	0.21
No. of cases of gestational diabetes	0.03
Hormonal contraceptives	0.14
Cause of amenorrhea	0.15
Menopause treatment	0.01
Weight (Kg)	0.16
Height (cm)	0.15
Waist (cm)	0.15
Hip (cm)	0.12
No. times eating outside /week	0.25
Vitamin supplements	0.07
Sweeteners intake	0.14
Fructose intake (y/n)	0.02
Types of salt for cooking	0.23
No. of meals /day	0.26
Snaks between meals	0.24
Eat while watching TV	0.2
Fruit and nat. juice intake/month	0.23
Nuts intake/month	0.19
Fresh fish & seafood intake/month	0.18
Canned fish & seafood intake/month	0.21
Meat intake/month	0.18
Cold meats intake/month	0.24
Alcohol intake frequency	0.27
Sugar soft-drinks /month	0.16
Sugar-free soft-drinks	0.08
Packaged juice /month	0.15
Vigorous physical act. (min/week)	0.06
Moderate exercise min/week	0.13
Walking minutes per week	0.16
Studies	0.23
BMI (Kg/m2)	0.15
Waist to Hip Ratio	0.16
Patient takes IECA	0.05
Patient takes ARA-II	0.04
Takes beta-blockers	0.03
Calcium channel blockers	0.02
Patient takes diuretics	0.05
Takes alpha-blockers	0.0
Renin inhibitors	0.0
No. hypotensive drugs	0.11
Walking MET-minutes per week	0.19
Moderate MET-minutes /week	0.14
Vigorous MET-minutes /week	0.07
Total physical activity MET (min/week)	0.19
Workout hours per week	0.17
Sitting hours /day	0.18
Smoking Pack-Years	0.17
1st-degree blood relatives with T2D	0.17
2nd-degree blood relatives with T2D	0.16
3rd-degree blood relatives with T2D	0.1
Tea and other herbal infusions /month	0.15
Coffee intake/month	0.24
Beverages with caffeine /month	0.24
Wine intake/month	0.15
Vegetables and legumes intake/month	0.19
Fried & precooked food intake/month	0.21
Eggs and dairy /month	0.19
Bread, rice, pasta intake/month	0.16
% Whole grain products	0.16
Sugar snacks and desserts /month	0.22
High-fat sauces & accompaniments /month	0.21
Changes to improve diet	0.04
Beer intake/month	0.16
Types of oil for cooking	0.13

4 Software

In this study, we have used Python 3.8.10 together with widely used libraries for data preprocessing, analysis, visualization and machine learning. The main libraries used, along with their specific versions and purposes, are listed in Table S11.

Table S11. Main Python libraries used in this work.

**Name**	**Version**	**Purpose**
Numpy	1.22.4	General math computing
Pandas	2.0.3	Data structuring, preprocessing
Scipy	1.10.1	Data analysis
Matplotlib	3.4.3	Visualization
Missingno	0.5.2	Visualization (missing data)
Scikit-learn	1.3.2	Machine learning, imputation
Optuna	3.6.1	Hyperparameter optimization
XGBoost	2.0.3	Machine learning (classification)

5 Model hyperparameters

As explained in the manuscript, a hyperparameter optimization was performed for each model through a 20-fold cross-validation based search, using Tree Parzen Estimator. Table S12 summarizes the hyperparameter search spaces explored during the optimization process.

Table S12. Hyperparameter search spaces explored for each XGBoost model.

Parameter	Search space
booster	[gbtree]
objective	[binary:logistic]
n_estimators	50 — 200
learning_rate	0.001 — 1
max_depth	1 — 10
grow_policy	[depthwise, lossguide]
subsample	0.8 — 1
colsample_bytree	0.5 — 1
colsample_bylevel	0.5 — 1
colsample_bynode	0.5 — 1
reg_alpha	0.0001 — 1
reg_lambda	0.0001 — 1
scale_pos_weight	1 — 20

Following hyperparameter optimization, models were trained for each scenario using the best hyperparameter combinations identified. The optimized hyperparameters for the models corresponding to each scenario are presented in Table S13.

Table S13. Optimized hyperparameters for the four proposed models using TPE with Optuna.

Parameter	D-ENV	D-HEA	P-ENV	P-HEA
booster	gbtree			
objective	binary:logistic			
n_estimators	136	99	181	103
learning_rate	0.12	0.12	0.05	0.01
max_depth	13	2	9	6
grow_policy	lossguide	depthwise		
subsample	0.98	0.56	0.85	0.62
colsample_bytree	0.71	0.55	0.50	0.98
colsample_bylevel	1	0.93	0.76	0.70
colsample_bynode	0.50	0.90	0.53	0.67
reg_alpha	0.04	0.01	0.13	0
reg_lambda	0	5.8	1.08	0.98
scale_pos_weight	8.44	6.91	7.33	4.30
